# Resolutions on the Death of Dr. W. W. Allport

**Published:** 1893-07

**Authors:** 


### Resolutions on the Death of Dr. W. W. Allport.

RESOLUTIONS OF THE NEBRASKA STATE DENTAL SOCIETY ON THE DEATH OF DR. W. W. ALLPORT.

WHEREAS, This society has learned with sorrow of the death of Dr. W. W. Allport, of Chicago, and of the loss sustained by the profession at large. Therefore, be it

*Resolved*, That as Dr. Allport was a man of high and lofty principles, ever ready to help the weak, quick to detect fraud, ever right in his dealings with his fellowmen, high and exalted in his attainments in the profession of his early adoption, it is but right and just that we, the members of this society, express our regard for one whose labors were confined not to the State and city in which he lived, but were as broad as his calling.

*Resolved*, That we tender our heartfelt sympathy to the family of the deceased, and that a copy of these resolutions be sent to the family of the deceased.

W. C. Davis, *Corresponding Secretary.*

---

RESOLUTIONS ADOPTED BY THE ODONTOLOGICAL SOCIETY OF CHICAGO.

Whereas, In the death of Dr. W. W. Allport, a leader in our profession has fallen and as a mark of our appreciation of his services and skill, be it

*Resolved*, That in his death the dental profession has lost a member whose extraordinary skill as an operator placed him among the foremost dentists of the world. His work in promoting the highest interests of the profession will ever be conspicuous and the prosperity enjoyed by younger members is due in a great measure to his achievements.

*Resolved*, That a copy of this preample and resolutions be sent in proper form to the family of the deceased and to the dental journals for publication.

Truman W. Brophy,
A. W. Harlan,
F. H. Gardiner.

---

RESOLUTIONS ADOPTED BY THE CHICAGO DENTAL SOCIETY, TUESDAY EVENING, JUNE 6TH, 1893, ON THE DEATH OF DR. W. W. ALLPORT.

Whereas, In the death of Dr. W. W. Allport, a leader in our profession has fallen, and as a mark of our appreciation of his services and skill, be it

*Resolved*, That in his death the dental profession has lost a member whose extraordinary skill as an operator placed him among the foremost dentists of the world; his work in promoting the highest interests of the profession will ever be conspicuous, and the prosperity enjoyed by the younger members is due in a great measure to his achievements.

*Resolved*, That a copy of this preamble and resolutions be sent in proper form to the family of the deceased and also to the dental journals for publication.

TRUMAN W. BROPHY,

A. W. HARLAN,

J. N. CROUSE,

*Committee.*

